# Is staff consistency important to parents’ satisfaction in a longitudinal study of children at risk for type 1 diabetes: the TEDDY study

**DOI:** 10.1186/s12902-021-00929-w

**Published:** 2022-01-10

**Authors:** Jessica Melin, Kristian F. Lynch, Markus Lundgren, Carin Andrén Aronsson, Helena Elding Larsson, Suzanne Bennett Johnson, TEDDY Study Group, Marian Rewers, Aaron Barbour, Kimberly Bautista, Judith Baxter, Daniel Felipe-Morales, Brigitte I. Frohnert, Marisa Stahl, Patricia Gesualdo, Rachel Haley, Michelle Hoffman, Rachel Karban, Edwin Liu, Alondra Munoz, Jill Norris, Stesha Peacock, Hanan Shorrosh, Andrea Steck, Megan Stern, Kathleen Waugh, Jorma Toppari, Olli G. Simell, Annika Adamsson, Sanna-Mari Aaltonen, Suvi Ahonen, Mari Åkerlund, Leena Hakola, Anne Hekkala, Henna Holappa, Heikki Hyöty, Anni Ikonen, Jorma Ilonen, Sanna Jokipuu, Leena Karlsson, Jukka Kero Miia Kähönen, Mikael Knip, Minna-Liisa Koivikko, Katja Kokkonen, Merja Koskinen, Mirva Koreasalo, Kalle Kurppa, Salla Kuusela, Jarita Kytölä, Sinikka Lahtinen, Jutta Laiho, Tiina Latva-aho, Laura Leppänen, Katri Lindfors, Maria Lönnrot, Elina Mäntymäki, Markus Mattila, Maija Miettinen, Katja Multasuo, Teija Mykkänen, Tiina Niininen, Sari Niinistö Mia Nyblom, Sami Oikarinen, Paula Ollikainen, Zhian Othmani, Sirpa Pohjola, Jenna Rautanen, Anne Riikonen, Minna Romo, Satu Simell, Aino Stenius, Päivi Tossavainen, Mari Vähä-Mäkilä, Eeva Varjonen, Riitta Veijola, Irene Viinikangas, Suvi M. Virtanen, Jin-Xiong She, Desmond Schatz, Diane Hopkins, Leigh Steed, Jennifer Bryant, Katherine Silvis, Michael Haller, Melissa Gardiner, Richard McIndoe, Ashok Sharma, Stephen W. Anderson, Laura Jacobsen, John Marks, Anette G. Ziegler, Ezio Bonifacio, Cigdem Gezginci, Anja Heublein, Eva Hohoff, Sandra Hummel, Annette Knopff, Charlotte Koch, Sibylle Koletzko, Claudia Ramminger, Roswith Roth, Jennifer Schmidt, Marlon Scholz, Joanna Stock, Katharina Warncke, Lorena Wendel, Christiane Winkler, Helmholtz Zentrum München, Forschergruppe Diabetes, Åke Lernmark, Daniel Agardh, Carin Andrén Aronsson, Maria Ask, Rasmus Bennet, Corrado Cilio, Susanne Dahlberg, Malin Goldman Tsubarah, Emelie Ericson-Hallström, Annika Björne Fors, Lina Fransson, Thomas Gard, Monika Hansen, Susanne Hyberg, Berglind Jonsdottir, Helena Elding Larsson, Marielle Lindström, Markus Lundgren, Marlena Maziarz, Maria Månsson Martinez, Jessica Melin, Zeliha Mestan, Caroline Nilsson, Yohanna Nordh, Kobra Rahmati, Anita Ramelius, Falastin Salami, Anette Sjöberg, Carina Törn, William A. Hagopian, Michael Killian, Claire Cowen Crouch, Jennifer Skidmore, Christian Chamberlain, Brelon Fairman, Arlene Meyer, Jocelyn Meyer, Denise Mulenga, Nole Powell, Jared Radtke, Shreya Roy, Davey Schmitt, Sarah Zink, Dorothy Becker, Margaret Franciscus, Mary Ellen Dalmagro-Elias Smith, Ashi Daftary, Mary Beth Klein, Chrystal Yates, Jeffrey P. Krischer, Rajesh Adusumali, Sarah Austin-Gonzalez, Maryouri Avendano, Sandra Baethke, Brant Burkhardt, Martha Butterworth, Nicholas Cadigan, Joanna Clasen, Kevin Counts, Christopher Eberhard, Steven Fiske, Laura Gandolfo, Jennifer Garmeson, Veena Gowda, Belinda Hsiao, Christina Karges, Qian Li, Shu Liu, Xiang Liu, Kristian Lynch, Jamie Malloy, Cristina McCarthy, Jose Moreno, Hemang M. Parikh, Cassandra Remedios, Chris Shaffer, Susan Smith, Noah Sulman, Roy Tamura, Dena Tewey, Michael Toth, Ulla Uusitalo, Kendra Vehik, Ponni Vijayakandipan, Melissa Wroble, Jimin Yang, Kenneth Young, Michael Abbondondolo, Lori Ballard, Rasheedah Brown, David Cuthbertson, Stephen Dankyi, David Hadley, Kathleen Heyman, Francisco Perez Laras, Hye-Seung Lee, Colleen Maguire, Wendy McLeod, Aubrie Merrell, Steven Meulemans, Ryan Quigley, Laura Smith, Beena Akolkar, Thomas Briese, Todd Brusko, Bennett Johnson, Eoin McKinney, Tomi Pastinen

**Affiliations:** 1 Department of Clinical Sciences, Lund University, Malmö, Sweden; 2 Health Informatics Institute, Morsani College of Medicine, University of South Florida, Tampa, FL USA; 3 Department of Pediatrics, Kristianstad hospital, Kristianstad, Sweden; 4 Department of Pediatrics, Skåne University Hospital, Malmö, Sweden; 5 Department of Behavioral Sciences and Social Medicine, Florida State University College of Medicine, Tallahassee, FL USA

**Keywords:** Study satisfaction, Parent satisfaction, Staff consistency, Child, Longitudinal study, Type 1 diabetes, Genetic risk

## Abstract

**Background:**

Participants’ study satisfaction is important for both compliance with study protocols and retention, but research on parent study satisfaction is rare. This study sought to identify factors associated with parent study satisfaction in The Environmental Determinants of Diabetes in the Young (TEDDY) study, a longitudinal, multinational (US, Finland, Germany, Sweden) study of children at risk for type 1 diabetes. The role of staff consistency to parent study satisfaction was a particular focus.

**Methods:**

Parent study satisfaction was measured by questionnaire at child-age 15 months (5579 mothers, 4942 fathers) and child-age four years (4010 mothers, 3411 fathers). Multiple linear regression analyses were used to identify sociodemographic factors, parental characteristics, and study variables associated with parent study satisfaction at both time points.

**Results:**

Parent study satisfaction was highest in Sweden and the US, compared to Finland. Parents who had an accurate perception of their child’s type 1 diabetes risk and those who believed they can do something to prevent type 1 diabetes were more satisfied. More educated parents and those with higher depression scores had lower study satisfaction scores. After adjusting for these factors, greater study staff change frequency was associated with lower study satisfaction in European parents (mothers at child-age 15 months: − 0.30,95% Cl − 0.36, − 0.24, *p* < 0.001; mothers at child-age four years: -0.41, 95% Cl − 0.53, − 0.29, *p* < 0.001; fathers at child-age 15 months: -0.28, 95% Cl − 0.34, − 0.21, *p* < 0.001; fathers at child-age four years: -0.35, 95% Cl − 0.48, − 0.21, *p* < 0.001). Staff consistency was not associated with parent study satisfaction in the US. However, the number of staff changes was markedly higher in the US compared to Europe.

**Conclusions:**

Sociodemographic factors, parental characteristics, and study-related variables were all related to parent study satisfaction. Those that are potentially modifiable are of particular interest as possible targets of future efforts to improve parent study satisfaction. Three such factors were identified: parent accuracy about the child’s type 1 diabetes risk, parent beliefs that something can be done to reduce the child’s risk, and study staff consistency. However, staff consistency was important only for European parents.

**Trial registration:**

NCT00279318.

## Background

Clinical trials are necessary to test various drugs as well as other medical or behavioral interventions. Natural history studies are critical to our understanding of disease etiology and progression. Both place considerable demands on participants who often endure invasive interventions or assessment procedures over long periods of time. Participants’ satisfaction with a trial or study experience is likely important to both compliance with the study protocol and study retention. However, few studies have examined study satisfaction and experiences among study participants [1]. This dearth of literature is particularly noteworthy in pediatric populations. Published studies of parent satisfaction focus mostly on the child’s care or specific aspects of a study [2–6], but studies of parents’ overall satisfaction with a study in which their child is enrolled are limited. The studies that do exist suggest that most participating parents will recommend the study to others and are willing to participate in a new study [2, 5, 7].

Studies examining factors associated with parent study satisfaction are also limited. Less educated parents and those from racial or ethnic minority groups often report greater satisfaction than higher educated parents or those from the majority culture [3, 8]. Higher overall satisfaction was also associated with fewer transportation problems and fewer study-related financial difficulties [6]. Most studies examining parent satisfaction do not report differences between mothers’ and fathers’ satisfaction; studies examining fathers’ study satisfaction are rare.

Type 1 diabetes is an autoimmune disease usually diagnosed in childhood; its prevalence is increasing worldwide [9]. Exogenous insulin replacement by injection or insulin pump is necessary for survival and there is no cure for type 1 diabetes. Although children at risk for type 1 diabetes can be identified by genetic and immunologic markers, there currently is no means to prevent the disease in these high-risk children. The Diabetes Prevention Trial (DPT-1) tested both insulin injections and oral insulin as possible prevention strategies in children at risk for type 1 diabetes; neither intervention was effective [10, 11]. Participants’ satisfaction was examined in all arms of the study. There was a high level of study satisfaction overall, but some important differences between participants emerged: parents reported greater study satisfaction than children, adult participants reported greater study satisfaction than child participants, and mothers reported greater study satisfaction than fathers [12, 13].

The Environmental Determinants of Diabetes in the Young (TEDDY) study was designed to identify environmental triggers of type 1 diabetes in genetically at-risk children from birth to 15 years of age. Using the DPT-1 methodology, TEDDY monitors parent study satisfaction on an ongoing basis and has found it to be associated with both study retention [14] and adherence with OGTT assessments [15], but not food record compliance [16].

The role of study staff as a determinant of participants’ study satisfaction seems critical since it is a potentially modifiable component of a study’s protocol. Studies suggest that participants were more likely to report greater study satisfaction when they felt the study staff had enough time for them, listened to them, and treated them with respect and friendliness [5, 17–19]. Few studies have examined whether consistency in study staff is important to participants’ study satisfaction. In a study examining reasons for retention, 89% of the parents gave the highest rating “liked a lot” for “seeing the same staff at each visit” [20]. Another study reported frequent staff changes across study visits as one of the top three most negative experiences for participants in a long-term trial [21].

The aim of the current study was to identify factors associated with parent study satisfaction in TEDDY, with a particular focus on the role of staff consistency. The study is unique in that it examines study satisfaction in both mothers and fathers in a multinational study at two points in time: after one year and after 4 years. Furthermore, the availability of psychosocial and sociodemographic variables collected during the TEDDY study permitted an examination of a wide range of factors potentially associated with study satisfaction. Since the different TEDDY countries have different approaches to study staffing, TEDDY data provided an important opportunity to examine the association of staff consistency with parent study satisfaction.

## Methods

### The TEDDY study

The aim of the TEDDY study is to identify environmental triggers of diabetes-related autoimmunity and progression to type 1 diabetes in genetically at-risk children. A total of 8676 children were enrolled in TEDDY before 4.5 months of age. Enrollment occurred during 2004–2010 in four different countries: Finland, Germany, Sweden, and the US. Each country’s ethical board approved the TEDDY study [22]. Most participants (89%) have no family members with type 1 diabetes. TEDDY children are followed until type 1 diabetes diagnosis or 15 years of age. The protocol includes four visits per year until four years of age, with visits reduced to two times per year for islet autoantibody-negative children while islet autoantibody-positive children maintain quarterly visits. Study visits include data collection from interviews, questionnaires, blood draws, and nasal swabs.

### Study satisfaction

In TEDDY, overall study satisfaction was measured by questionnaire at a child’s 6- and 15-months visits, then annually thereafter. In the present study, we used the data from the 15-month and the four-year questionnaires, since 15 months is one year after enrollment and four years is the end of the quarterly visit schedule for all TEDDY children. Both mothers and fathers were administered the questionnaires. Using the same methodology employed in the DPT-1 [12, 13], study satisfaction was measured by three items: 1) “Overall, how do you feel about having your child participate in the TEDDY study? (scored 2 =like it a lot, 1 =like it a little, 0 =it is ok or dislike it),” 2) “Do you think your child’s participation in TEDDY was a good decision? (scored: 2 = a great decision, 1 = a good decision, 0= an ok decision or bad decision)’” and 3) “Would you recommend the TEDDY study to a friend? (scored: 2 =yes, 1 =maybe, 0 =no).” Since these items were highly correlated, they were summed to create a total satisfaction score with a range of 0–6. Reliability estimates for this sample ranged from α = 0.80 to α = 0.83.

### Study sample/population

**Fig. 1:**
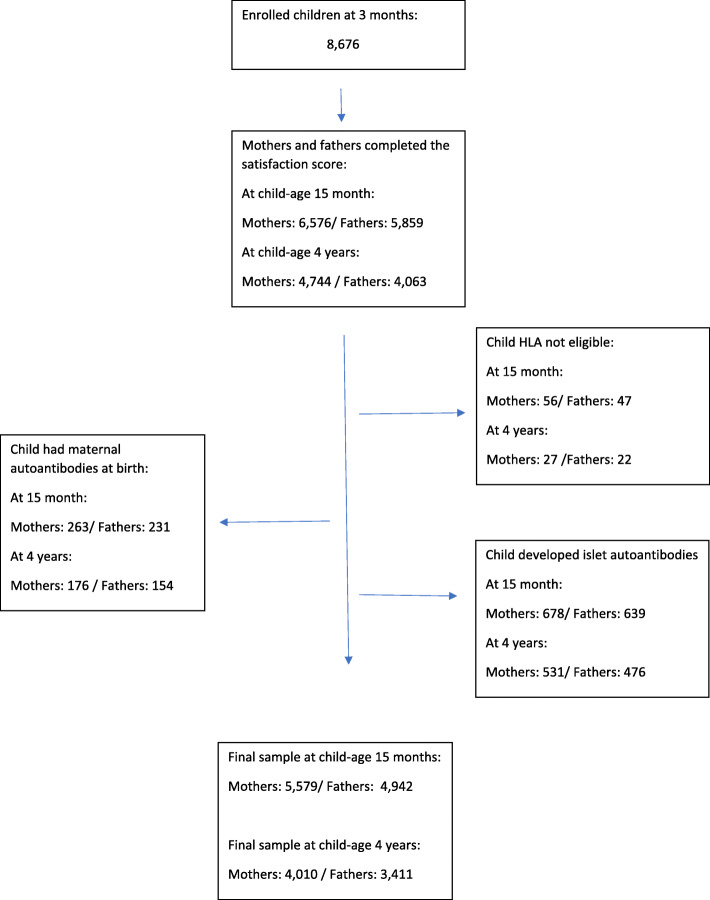
Flowchart

We examined study satisfaction at two different time points: one year (child-age 15 months) and four years after enrollment (child-age 48 months). Out of the 8676 enrolled children, there were 6576 mothers and 5859 fathers at child-age 15 months who had completed the study satisfaction measure. At child-age four years, there were 4744 mothers and 4063 fathers with study satisfaction scores. Parents were excluded if the child was not HLA eligible (child-age 15 months: 56 mothers and 47 fathers; child-age four years: 27 mothers and 22 fathers); the child had maternal autoantibodies at birth (child-age 15 months: 263 mothers and 231 fathers; child-age four years: 176 mothers and 154 fathers; or the child developed islet autoantibodies for type 1 diabetes (child-age 15 months: 678 mothers and 639 fathers; child-age four years: 531 mothers and 476 fathers). The final sample consisted of 5579 mothers and 4942 fathers at child-age 15 months and 4010 mothers and 3411 fathers at child-age four years (Fig. 1).

### Sociodemographic measures

Sociodemographic variables collected at study inception included country of residence, child sex, child has a first-degree relative (mother, father, or sibling) with type 1 diabetes (yes/no), parental age at birth of the child, and child ethnic minority status (yes = for USA: the mother was not born in the USA, the mother’s first language is not English, or the child is a member of an ethnic minority; for Europe: the country of birth or mother’s first language is one other than that of the TEDDY country in which the child is living. Others = no). Additional sociodemographic variables collected at the 9-month visit included parent education (categorized as primary education or some trade school, graduated trade school, graduated college/university or higher), parent’s first child (yes/no), and parent’s marital status (parents married/living together: yes/no).

### Study-related variables

#### Recruitment cohort

The recruitment cohort was of interest because TEDDY made a protocol change in March 2009 which introduced a study intervention designed to reduce study drop-outs [23]. Consequently, two recruitment cohorts were examined: children enrolled in TEDDY from September 2004–February 2009 and children enrolled from March 2009–February 2010.

#### Study staff consistency

Since all personnel working in the TEDDY study have a specific staff code recorded at every study visit, the number of staff changes prior to any given study visit could be examined. For the 15-month analysis, we counted the number of staff changes for that specific family from enrollment to the 15-month visit (from a minimum of 0 to a maximum of 4). For the four-year analysis, we counted the number of staff changes in the year prior to the four-year visit (from a minimum of 0 to a maximum of 3). We also examined the total number of staff changes from study inception to the four-year study visit. However, the number of staff changes in the year prior to the four-year visit proved to be more strongly related to parent study satisfaction and is presented here.

### Parent lifestyle behaviors

At the 9-month study visit, the following parent lifestyle behaviors were collected: smoking (yes/no) and working outside the home (yes/no).

### Parent depression

The Depression subscale of the Well Being Questionnaire^1^ [24] was collected at the 15-month visit, than annually thereafter, with higher scores indicating a higher level of depression. The scores obtained at the 15-month and the four-year visit were used in the current analysis. Reliability estimates for this sample ranged from α = 0.62 to α = 0.69.

### Parent reactions to the TEDDY Child’s type 1 diabetes risk

Parent reactions to the child’s type 1 diabetes risk were assessed at six months, 15 months and annually thereafter. Data collected at the 15-month and four-year visit were used in the current analysis.

#### Risk perception accuracy

The accuracy of the parent’s perception of the child’s risk for type 1 diabetes was assessed by the following item: “Compared to other children, do you think your child’s risk for developing diabetes is: much lower, somewhat lower, about the same, somewhat higher, or much higher. Responses of “higher” or “much higher” were categorized as accurate, while all other responses were categorized as inaccurate.

#### Belief that the Child’s type 1 diabetes risk can be reduced

Two questions were used to assess parent beliefs that the child’s diabetes risk can be reduced: “I can do something to reduce my child’s risk of developing diabetes” and “Medical professionals can do something to reduce my child’s risk for developing diabetes.” The parent was asked to agree or disagree with each statement on a five-point scale, from strongly agree to strongly disagree. The two items were reverse scored and summed to create a total score with a range of 0–8. A higher score indicated greater belief that the child’s risk of type 1 diabetes can be reduced. The coefficient alpha for this sample ranged from α = 0.67 to α = 0.71.

#### Parent anxiety about the Child’s risk for type 1 diabetes

Parent anxiety about the child’s risk for type 1 diabetes was measured by a 6-item short form [25] of the State Anxiety component of The State-Trait-Anxiety Inventory for Adults™ (STAI)^2^ [26]. The measure was designed to specifically assess the parent’s anxiety about the child’s risk for type 1 diabetes. The 6-item short form score was converted to the 20-item scale score to make it comparable with the larger STAI literature [25]; a higher score indicated higher anxiety. Reliability estimates for this sample ranged from α = 0.90 to α = 0.91.

### Data analysis

Comparison of study variables across independent groups were conducted using ANOVA for continuous variables and chi-square for categorical variables. Paired t-tests were used to compare mothers’ and fathers’ satisfaction scores. Multiple linear regression models were used to identify factors associated with study satisfaction of mothers and fathers separately, when the child was 15 months and four years of age. The analysis was done block by block in the following order: sociodemographic, study-related variables, parent lifestyle behaviors, parent depression, and parent reaction to the child’s type 1 diabetes risk. At each step, variables with a *p* value of < 0.10 were retained. The final model included all variables with a *p* value of < 0.05 in either the mother or father models. Since staff change was markedly higher in the US than Europe, the interaction of staff change by Europe (yes/no) was tested in all final models.

SPSS version 27 (IBM SPSS Statistics for Windows, Armonk, NY: IBM Corp) were used for the statistical analyses.

## Results

Descriptive statistics for all study variables at child-age 15 months are provided by country for mothers (Table 1) and fathers (Table 2). There were significant country differences for all study variables except child sex and parents living together (fathers only). Noteworthy is the large difference in the number of staff changes in the US compared to Europe. In the first 15 months of the study, there were an average of 2.5 staff changes in the US compared to an average of 1.1 staff changes in Finland and 0.3 staff changes in Sweden (mothers’ data), a highly significant difference (*p* < 0.001 both for mothers and fathers).

**Table 1:** Mothers’ sample characteristics at child-age 15 months by country

Variable	US *n* = 2269	Finland *n* = 1218	Germany *n* = 303	Sweden *n* = 1794	*p* value
	*n* (%) or mean (SD)	*n* (%) or mean (SD)	*n* (%) or mean (SD)	*n* (%) or mean (SD)	
**Demographics:**					
Child sex:					0.817
Female	1112 (49.0)	598 (49.1)	142 (46.9)	893 (49.8)	
Male	1157 (51.0)	620 (50.9)	161 (53.1)	901 (50.2)	
First-degree relative with type 1 diabetes:					< 0.001
No	2081 (91.7)	1144 (93.9)	205 (67.7)	1706 (95.1)	
Yes	188 (8.3)	74 (6.1)	98 (32.3)	88 (4.9)	
Child ethnic minority:					< 0.001
No	1596 (70.3)	1139 (93.5)	252 (83.2)	1648 (91.9)	
Yes	608 (26.8)	33 (2.7)	40 (13.2)	113 (6.3)	
Missing	65 (2.9)	46 (3.8)	11 (3.6)	33 (1.8)	
First child:					< 0.001
No	1385 (61.0)	662 (54.4)	156 (51.5)	977 (54.5)	
Yes	856 (37.7)	536 (44.0)	136 (44.9)	804 (44.8)	
Missing	28 (1.2)	20 (1.6)	11 (3.6)	13 (0.7)	
Parents living together:					< 0.001
No	116 (5.1)	34 (2.8)	7 (2.3)	39 (2.2)	
Yes	2125 (93.7)	1166 (95.7)	285 (94.1)	1741 (97.0)	
Missing	28 (1.2)	18 (1.5)	11 (3.6)	14 (0.8)	
Mother’s education:					< 0.001
University	1412 (62.2)	723 (59.4)	111 (36.6)	879 (49.0)	
Trade School	513 (22.6)	371 (30.5)	145 (47.9)	310 (17.3)	
Basic Primary	329 (14.5)	104 (8.5)	36 (11.9)	594 (33.1)	
Missing	15 (0.7)	20 (1.6)	11 (3.6)	11 (0.6)	
Mother’s age at child’s birth	2269	1218	303	1794	< 0.001
	31.0 (5.5)	30.1 (4.8)	32.1 (4.8)	31.0 (4.6)	
**Study variables:**					
Number of staff member changes:	2141	1178	253	1759	< 0.001
	2.5 (1.3)	1.1 (1.3)	0.8 (0.8)	0.3 (0.6)	
Recruitment cohort:					< 0.001
Sept 2004-Feb 2009	1710 (75.4)	1005 (82.5)	243 (80.2)	1446 (80.6)	
Mar 2009-Feb 2010	559 (24.6)	213 (17.5)	60 (19.8)	348 (19.4)	
**Lifestyle variables:**					
Mother smokes:					< 0.001
No	2097 (92.4)	1068 (87.7)	254 (83.8)	1602 (89.3)	
Yes	146 (6.4)	133 (10.9)	38 (12.5)	181 (10.1)	
Missing	26 (1.1)	17 (1.4)	11 (3.6)	11 (0.6)	
Mother works outside home:					< 0.001
No	981 (43.2)	1018 (83.6)	231 (76.2)	1282 (71.5)	
Yes	1248 (55.0)	175 (14.4)	60 (19.8)	497 (27.7)	
Missing	40 (1.8)	25 (2.1)	12 (4.0)	15 (0.8)	
**Depression subscale:**	2266	1218	303	1793	< 0.001
	2.8 (2.2)	2.8 (2.1)	2.7 (2.4)	3.8 (2.2)	
**Maternal reaction to child’s type 1 diabetes risk:**					
Risk perception:					< 0.001
Underestimate	934 (41.2)	354 (29.1)	88 (29.0)	831 (46.3)	
Accurate	1331 (58.7)	863 (70.9)	213 (70.3)	962 (53.6)	
Missing	4 (0.2)	1 (0.1)	2 (0.7)	1 (0.1)	
Anxiety (STAI):	2257 35.5 (10.6)	1218 30.7 (7.8)	299 36.6 (10.5)	1787 34.0 (8.6)	< 0.001
Belief that child’s type 1 diabetes risk can be reduced:	2262 4.4 (1.8)	1218 4.8 (1.7)	303 4.4 (1.9)	1793 5.2 (1.4)	< 0.001

Mothers Excluded: children not HLA eligible, children with positive islet autoantibodies, children with maternal islet autoantibodies at 3 or 6 months and children with no maternal study satisfaction measure at 15 months

**Table 2:** Fathers’ sample characteristics at child-age 15 months by country

Variable	US *n* = 1853		Finland *n* = 1152	Germany *n* = 285	Sweden *n* = 1666	*p* value
	*n* (%) or mean (SD)		*n* (%) or mean (SD)	*n* (%) or mean (SD)	*n* (%) or mean (SD)	
**Demographics:**						
Child sex:						0.872
Female	902 (48.7)		565 (49.0)	135 (47.4)	828 (49.7)	
Male	951 (51.3)		587 (51.0)	150 (52.6)	838 (50.3)	
First-degree relative with type 1 diabetes:						< 0.001
No	1686 (91.0)		1079 (93.7)	194 (68.1)	1584 (95.1)	
Yes	167 (9.0)		73 (6.3)	91 (31.9)	82 (4.9)	
Child ethnic minority:						< 0.001
No	1349 (72.8)		1080 (93.8)	240 (84.2)	1536 (92.2)	
Yes	454 (24.5)		30 (2.6)	35 (12.3)	104 (6.2)	
Missing	50 (2.7)		42 (3.6)	10 (3.5)	26 (1.6)	
First child:						< 0.001
No	1116 (60.2)		616 (53.5)	143 (50.2)	888 (53.3)	
Yes	721 (38.9)		518 (45.0)	132 (46.3)	768 (46.1)	
Missing	16 (0.9)		18 (1.5)	10 (3.5)	10 (0.6)	
Parents living together:						0.295
No	25 (1.3)		16 (1.4)	1 (0.4)	15 (0.9)	
Yes	1810 (97.7)		1120 (97.2)	274 (96.1)	1640 (98.4)	
Missing	18 (1.0)		16 (1.4)	10 (3.5)	11 (0.7)	
Father’s education:						< 0.001
University	1116 (60.2)		590 (51.2)	109 (38.2)	647 (38.8)	
Trade School	378 (20.4)		419 (36.4)	131 (46.0)	281 (16.9)	
Basic Primary	326 (17.6)		101 (8.8)	33 (11.6)	721 (43.3)	
Missing	33 (1.8)		42 (3.6)	12 (4.2)	17 (1.0)	
Father’s age at child’s birth	1820		1132	284	1656	< 0.001
	33.6 (5.9)		32.3 (5.8)	35.3 (5.3)	33.5 (5.4)	
**Study variables:**						
Number of staff member changes:	1770		1115	240	1640	< 0.001
	2.6 (1.3)		1.1 (1.3)	0.8 (0.8)	0.3 (0.7)	
Recruitment cohort:						< 0.001
Sept 2004-Feb 2009	1391 (75.1)		952 (82.6)	227 (79.6)	1337 (80.3)	
Mar 2009-Feb 2010	462 (24.9)		200 (17.4)	58 (20.4)	329 (19.7)	
**Lifestyle variables:**						
Father smokes:						< 0.001
No	1653 (89.2)		844 (73.3)	209 (73.3)	1481 (88.9)	
Yes	182 (9.8)		284 (24.7)	66 (23.2)	175 (10.5)	
Missing	18 (1.0)		24 (2.1)	10 (3.5)	10 (0.6)	
Father works outside home:						0.006
No	121 (6.5)		103 (8.9)	31 (10.9)	151 (9.1)	
Yes	1693 (91.4)		997 (86.5)	244 (85.6)	1503 (90.2)	
Missing	39 (2.1)		52 (4.5)	10 (3.5)	12 (0.7)	
**Depression subscale:**	1849		1151	284	1665	< 0.001
	2.3 (2.0)		2.1 (1.8)	2.0 (2.1)	3.0 (1.9)	
**Paternal reaction to child’s type 1 diabetes risk:**						
Risk perception:						< 0.001
Underestimate	1025 (55.3)		533 (46.3)	104 (36.5)	895 (53.7)	
Accurate	825 (44.5)		619 (53.7)	180 (63.2)	770 (46.2)	
Missing	3 (0.2)		0 (0.0)	1 (0.3)	1 (0.1)	
Anxiety (STAI):	1841 33.1 (10.9)		1148 29.3 (7.7)	279 35.0 (10.7)	1654 31.7 (8.2)	< 0.001
Belief that child’s type 1 diabetes risk can be reduced:	1850 4.9 (1.8)		1151 5.2 (1.6)	284 4.6 (1.8)	1661 5.6 (1.4)	< 0.001

Fathers Excluded: children not HLA eligible, children with positive islet autoantibodies, children with maternal islet autoantibodies at 3 or 6 month and children with no father study satisfaction measure at 15 months

### Parent satisfaction score at 15-months and 4 years

The parent satisfaction score ranged from 0 to 6 and was skewed in a positive direction. At child-age 15-months, 45% of the mothers and 38% of the fathers had a score of six, the highest possible satisfaction score. At child-age four years, the results were similar with 48% of mothers and 40% of fathers with a score of six. Mothers had higher scores (M = 4.50, 95% Cl 4.46,4.55) than fathers (M = 4.12, 95% Cl 4.07, 4.17) at 15-months *p* < 0.001. The results were similar at four years (mothers: M = 4.59, 95% Cl 4.53,4.64; fathers: M = 4.18, 95% Cl 4.12, 4.25, *p* < 0.001). A total of 3825 mothers and 3138 fathers completed both the 15-month and four-year satisfaction measure. In this subgroup, parent satisfaction scores remained high over time (mothers: 15-months M = 4.68, 95% Cl 4.62, 4.73 and four-years M = 4.60, 95% Cl 4.55, 4.66; fathers: 15-months M = 4.25, 95% Cl 4.19, 4.31 and four-years M = 4.20, 95% Cl 4.13, 4.26).

### Variables associated with mothers’ study satisfaction

Table 3 describes the factors associated with mothers’ study satisfaction at child-age 15 months and four years. The results were similar at both time-points. Country, mother’s education level, parents living together, maternal depression, risk perception accuracy, anxiety about the child’s type 1 diabetes risk, and belief that the child’s type 1 diabetes risk could be reduced were all associated with mothers’ study satisfaction at 15 months and at four years. Swedish and US mothers were more satisfied than Finnish mothers. Mothers with basic primary or trade school education were more satisfied than mothers with a university degree. Mothers living with the child’s father were less satisfied than those living apart, and mothers with higher depression subscale scores were less satisfied than those with lower depression scores. Mothers who were accurate about their child’s type 1 diabetes risk, those who were less anxious about the risk, and those who believed something could be done to reduce the child’s risk were all more satisfied with the TEDDY study. Mothers who were older at the child’s birth reported lower satisfaction scores at 15 months (*p* = 0.001), but not at four years (*p* = 0.734). More frequent staff change was associated with less study satisfaction at 15 months (− 0.04, 95% Cl-0.08,0.003, *p* = 0.071), but not at four years (0.01, 95% Cl − 0.05, 0.07, *p* = 0.806), although the 15-month study result only approached significance.

**Table 3:** Final generalized liner models for mothers’ satisfaction at child-age 15 months and 4 years

Child-age 15 months:					Child-age 4 years:				
	*n*	B*	95% CI	*P* value		*n*	B*	95% CI	*P* value
Country:									
Finland	1156		Reference			799		Reference	
US	2089	1.31	1.16, 1.44	< 0.001		1474	1.58	1.43, 1.74	< 0.001
Germany	245	0.26	0.03, 0.48	0.025		155	0.46	0.18, 0.72	0.001
Sweden	1734	1.31	1.18, 1.44	< 0.001		1421	1.55	1.39, 1.67	< 0.001
First-degree relative with type 1 diabetes:									
No	4819		Reference			3537		Reference	
Yes	405	0.14	−0.03, 0.31	0.103		312	0.03	−0.16, 0.22	0.766
Mother’s education:									
University	2977		Reference			2291		Reference	
Trade School	1252	0.35	0.24, 0.46	< 0.001		840	0.42	0.29, 0.54	< 0.001
Basic Primary	995	0.42	0.29, 0.55	< 0.001		718	0.53	0.39, 0.68	< 0.001
Parents living together:									
No	180		Reference			112		Reference	
Yes	5044	−0.42	−0.66, −0.18	0.001		3737	− 0.45	− 0.74, − 0.15	0.003
Mother’s age at child’s birth:	5224	−0.02	− 0.03, − 0.01	0.001		3849	− 0.002	− 0.01, 0.01	0.734
Number of staff changes in the previous year:	5224	−0.04	−0.08, 0.003	0.071		3849	0.01	−0.05, 0.07	0.806
Mother smokes:									
No	4754		Reference			3566		Reference	
Yes	470	0.08	−0.07, 0.24	0.297		283	0.17	−0.02, 0.37	0.080
Maternal depression:	5224	−0.05	−0.07, − 0.03	< 0.001		3849	− 0.05	− 0.08, − 0.03	< 0.001
Mother’s perception of child’s type 1 diabetes risk:									
Underestimate	2061		Reference			1460		Reference	
Accurate	3163	0.18	0.09, 0.28	< 0.001		2389	0.27	0.16, 0.37	< 0.001
Maternal anxiety:	5224	−0.01	−0.01, − 0.002	0.010		3849	−0.01	−0.01, − 0.001	0.026
Maternal belief that child’s type 1 diabetes risk can be reduced:	5224	0.13	0.11 0.16	< 0.001		3849	0.11	0.08, 0.14	< 0.001

* = B is the linear model coefficient and is interpreted as difference in mean satisfaction compared to the reference group for categorical variables or difference in mean satisfaction per 1 unit change in parental measure for continuous variables when adjusting for all other variables in model as listed in table

### Variables associated with fathers’ study satisfaction

The multiple linear regression model results for fathers are provided in Table 4. Similar to the findings for mothers, the variables of country, education level, parental depression, risk perception accuracy, and belief that the child’s type 1 diabetes risk could be reduced were all associated with fathers’ study satisfaction. At 15 months (− 0.07, 95% Cl − 0.11, − 0.02, *p* = 0.007) but not at four years (− 0.02, 95% Cl − 0.09, 0.05, *p* = 0.531), fathers were less satisfied if there were greater staff changes since enrollment. There were two additional findings for fathers that did not occur for mothers: fathers with type 1 diabetes in the family were more satisfied (*p* = 0.006) at child-age 15 months, while fathers who smoked were more satisfied with their participation in TEDDY (*p* = 0.032) at child-age 4 years.

**Table 4:** Final generalized liner model for fathers’ satisfaction at child-age 15 months and 4 years

Child-age 15 months:					Child-age 4 year:			
	*n*	B*	95% CI	*P* value	*n*	B*	95% CI	*P* value
Country:								
Finland	1057		Reference		682		Reference	
US	1692	1.37	1.22, 1.53	< 0.001	1058	1.50	1.32, 1.68	< 0.001
Germany	226	0.92	0.66, 1.17	< 0.001	142	0.42	0.13, 0.72	0.005
Sweden	1590	1.42	1.27, 1.57	< 0.001	1235	1.38	1.21, 1.54	< 0.001
First-degree relative with type 1 diabetes:								
No	4197		Reference		2864		Reference	
Yes	368	0.27	0.08, 0.46	0.006	253	0.06	−0.15, 0.27	0.562
Father’s education:								
University	2325		Reference		1591		Reference	
Trade School	1141	0.11	−0.02, 0.23	0.100	746	0.33	0.19, 0.47	< 0.001
Basic Primary	1099	0.19	0.05, 0.33	0.007	780	0.52	0.37, 0.67	< 0.001
Parents living together:								
No	23		Reference		9		Reference	
Yes	4542	−0.57	−1.28, 0.13	0.112	3108	− 0.79	−1.82, 0.24	0.132
Father’s age at child’s birth:	4565	−0.002	−0.01, 0.01	0.611	3117	0.003	−0.01, 0.01	0.586
Number of staff changes in the previous year:	4565	−0.07	−0.11, − 0.02	0.007	3117	− 0.02	− 0.09, 0.05	0.531
Father smokes:								
No	3920		Reference		2724		Reference	
Yes	645	0.05	− 0.10, 0.20	0.534	393	0.19	0.02, 0.36	0.032
Paternal depression:	4565	−0.05	− 0.08, − 0.02	< 0.001	3117	− 0.03	− 0.06, − 0.01	0.019
Father’s perception of child’s type 1 diabetes risk:								
Underestimate	2352		Reference		1570		Reference	
Accurate	2213	0.48	0.34, 0.59	< 0.001	1547	0.19	0.08, 0.31	0.001
Paternal anxiety:	4565	0.01	0.00, 0.01	0.071	3117	0.003	−0.003, 0.01	0.349
Paternal belief that child’s type 1 diabetes risk can be reduced:	4565	0.20	0.17, 0.23	< 0.001	3117	0.08	0.04, 0.11	< 0.001

* = B is the linear model coefficient and is interpreted as difference in mean satisfaction compared to the reference group for categorical variables or difference in mean satisfaction per 1 unit change in parental measure for continuous variables when adjusting for all other variables in model as listed in table

### Interaction of staff consistency with European versus US sites

**Fig. 2:**
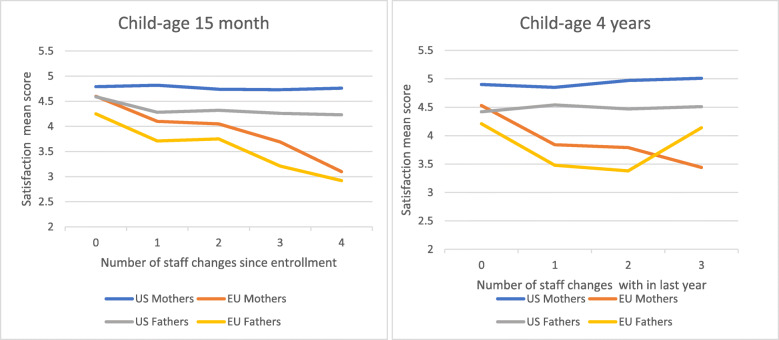
Study satisfaction at child-age 15 months and 4 years for US and Europe by number of staff changes in the last year

Because of the large differences in staff change frequency between European and US sites, we examined whether staff consistency was differentially important for Europe and the US. The interaction was significant for both mothers and fathers at both 15 months and four years. Consequently, we reran our final models for the US and Europe separately. Staff consistency was important for Europe, but not the US, with greater staff change frequency associated with lower study satisfaction among European mothers (child-age 15 months: − 0.30,95% Cl − 0.36, − 0.24, *p* < 0.001; child-age four years: -0.41, 95% Cl − 0.53, − 0.29, *p* < 0.001) and fathers (child-age 15 months: -0.28, 95% Cl − 0.34, − 0.21, *p* < 0.001; child-age four years: -0.35, 95% Cl − 0.48, − 0.21, *p* < 0.001). Adjusting for all other variables in the final model, one additional staff change before 15 months of age significantly decreased the mothers’ average satisfaction score by − 0.30 and fathers’ by − 0.28; at four years the score decreased by − 0.41 for mothers and − 0.35 for fathers. (Table 5, Fig. 2).

**Table 5:** Association of staff consistency with parent study satisfaction at child-age 15 months and 4 years

		US:							Europe:							
		*n*		B*		95% CI		*p*		*n*		B*		95% CI		*p*
**Numbers of staff changes at 15 months:**																
Mothers:		2089		0.01		−0,04, 0.06		0.591		3135		−0.30		−0.36, − 0.24		< 0.001
Fathers:		1692		−0.04		−0.11, 0.02		0.195		2873		−0.28		−0.34, − 0.21		< 0.001
**Number of staff changes at 4 years:**																
Mothers:		1474		0.06		−0.01, 0.13		0.085		2375		−0.41		−0.53, − 0.29		< 0.001
Fathers:		1058		0.02		−0.06, 0.10		0.626		2059		−0.35		−0.48, − 0.21		< 0.001

All variables included in Tables 3 and 4 are controlled

* = B is the linear model coefficient and is interpreted as difference in mean satisfaction compared to the reference group for categorical variables or difference in mean satisfaction per 1 unit change in parental measure for continuous variables when adjusting for all other variables in model as listed in table

## Discussion

Similar to prior studies of parent study satisfaction [2, 5–7, 21], most TEDDY parents were very satisfied with their study participation; over 45% of mothers and over 38% of fathers had the highest possible satisfaction score after 1 year and 4 years in the study. DPT-1 [12, 13] used the same questions to measure satisfaction as in this study and their results were similar to ours with high parental satisfaction among both mothers and fathers. In DPT-1, mothers were more satisfied than fathers [13]. Our study showed similar results: mothers’ mean satisfaction scores were higher than fathers’ at both 15 months and four years. Differences between mothers’ and fathers’ study satisfaction may be partially explained by mothers’ more active role in the study; although many fathers did participate, mothers more often accompanied the child to TEDDY visits.

The TEDDY study is unique in both its monitoring of parental study satisfaction across time and its comprehensive examination of factors potentially related to parent study satisfaction. Factors associated with study satisfaction were similar for mothers and fathers and showed a similar pattern at child-age 15 months and four years.

Like our study, others have reported that lower education level is associated with higher study satisfaction [3, 8]. Being part of a longitudinal study with visits several times per year provides a parent with an opportunity to ask questions and talk to professionals about the child’s type 1 diabetes risk. Perhaps this is more important to parents with lower education compared to more highly educated parents who may more readily access information elsewhere. The repeated study visits may also provide important support to mothers who are not living with the child’s father; these mothers reported greater satisfaction with their study participation.

We found that variables related to parental reactions to their child’s type 1 diabetes risk to be associated with study satisfaction. Both parents with accurate risk perception and parents who believed they could do something to prevent their child from developing type 1 diabetes had higher study satisfaction scores. Anxiety about the child’s type 1 diabetes risk showed a weaker association. More anxious mothers were less satisfied at both child-age 15 months and 4 years. Father anxiety was weakly associated with study satisfaction at 15 months, but not at 4 years. The accuracy of a parent’s perception of their child’s diabetes risk is a modifiable variable. Parents participating in the TEDDY study receive information about their child’s type 1 diabetes risk at least once a year. Knowing one’s child is at risk for a chronic disease may increase a parent’s anxiety but also their willingness to continue participating. Knowing that someone is watching for signs and symptom of type 1 diabetes in their child may provide some comfort and a greater sense of satisfaction with study participation.

The role of study staff in parent study satisfaction is likely influenced by the invasiveness, duration, and frequency of study visits. Previous research has shown that even in studies with a short duration, participants’ satisfaction increased when they felt study staff had time for them, listened to them, treated them with respect, and were easy to communicate with [5, 17, 19]. In long-term trials, the consistency of study staff may be particularly important [21]. Prior study participants have expressed feelings of abandonment when their staff were transferred or when the study ended without opportunities for future contact [19]. In our study, we found that staff consistency was associated with European parent study satisfaction at both child-age 15 months and after four years. For these parents, fewer changes in study staff were associated with higher satisfaction scores, although this was not the case among US parents. This result is consistent with a prior report which found that one of the reasons for Swedish families to stay in TEDDY was to be seen by the same staff at all visits [27]. Although the participating countries in TEDDY follow the same study protocol, the operation of study clinics varies markedly. It was more common in European sites for participants to have a dedicated staff person following them though the study. This may be one of the reasons why European parents reported lower study satisfaction when faced with increasing staff changes. In contrast, from study inception, participants in US sites often saw different study staff across visits and were less affected by staff changes. Differences in health care systems may also play a part. European participants were all part of universal health care systems in which most children are followed by the same nurse or pediatrician from birth until they start school. US families have a very different health care experience. Some may see the same pediatrician on a regular basis, but many do not.

The present study had several limitation. We only investigated the importance of staff consistency and did not examine other staff characteristics shown to be important for study satisfaction or study retention, such as staff friendliness or responsiveness to questions [20]. Other studies have suggested that study staff often underestimate the importance of their own role in participant study satisfaction and study retention [19, 20]. This study is also limited to those who participated in TEDDY for at least one year. Consequently, we do not know if our findings apply to parents who withdrew from TEDDY in the first year. Our study only involved children who were at-risk for type 1 diabetes but did not yet have the disease; whether our findings would be similar for parents of children with diabetes remains to be seen. Similarly, the TEDDY study offers no intervention to prevent the disease. As a consequence, intervention trials may identify different factors associated with parental study satisfaction. However, the association of parent study satisfaction to both study retention and compliance [14, 15, 19, 20], suggests that the identification of factors associated with parent study satisfaction is important to the design of pediatric research studies. This study has numerous strengths in this regard, including a large sample size from four different countries, assessment of both mothers’ and fathers’ study satisfaction across long periods of time, use of a reliable measure of study satisfaction, and a comprehensive analysis of multiple factors for possible association with parental study satisfaction.

## Conclusions

Sociodemographic factors, parental characteristics, and study-related variables were all related to parent study satisfaction. Those that are potentially modifiable are of particular interest as possible targets of future efforts to improve parent study satisfaction. Three such factors were identified: parent accuracy about the child’s type 1 diabetes risk, parent beliefs that something can be done to reduce the child’s risk, and study staff consistency. However, staff consistency was important only for European parents.

## Data Availability

The datasets generated and analyzed during the current study will be made available in the NIDDK Central Repository at https://repository.niddk.nih.gov/studies/teddy.

## Abbreviation

DPT-1: The Diabetes Prevention Trial
TEDDY: The Environmental Determinants of Diabetes in the Young

## Footnotes

1 The Well Being Questionnaire is licensed by Clare Bradley, HEALTH PSYCHOLOGY RESEARCH LIMITED, (“HPR”), 188 High Street, TW20 9ED, United Kingdom: www.healthpsychologyresearch.com

2 Copyright© 1968, 1977 by Charles D. Spielberger. State-Trait Anxiety Inventory for Adults™ requires license purchase and is a trademark of Mind Garden, Inc.
